# Suppression of Glomerulonephritis in NZB/NZW Lupus Prone Mice by Adoptive Transfer of *Ex Vivo* Expanded Regulatory T Cells

**DOI:** 10.1371/journal.pone.0006031

**Published:** 2009-06-24

**Authors:** Kenneth J. Scalapino, David I. Daikh

**Affiliations:** 1 Arthritis Section, San Francisco Veterans Affairs Medical Center, San Francisco, California, United States of America; 2 Division of Rheumatology, Department of Medicine, University of California San Francisco, San Francisco, California, United States of America; New York University School of Medicine, United States of America

## Abstract

Systemic lupus erythematosus (SLE) is an autoimmune disease of unknown cause characterized by expansion of autoreactive lymphocytes. Regulatory T cells (T_regs_) are a component of the normal immune system and contribute to the maintenance of peripheral tolerance. T_reg_ abnormalities have been associated with several autoimmune diseases and there is interest in the role of T_regs_ in SLE. We previously demonstrated that transfer of expanded CD4^+^CD25^+^CD62L^HI^ T_regs_ slows the development of lupus in (NZBxNZW)F_1_ (B/W) mice. However in the absence of T_reg_ specific surface antigens, cell purification remains a compromise between the breadth and purity of the population isolated. Importantly, purified populations always contain Foxp3^−^ effector T cells (T_effs_) that theoretically could exacerbate autoimmunity in the recipient. Here we explore the impact of transferring the more comprehensive, but less pure T_reg_ subset defined by CD4^+^CD25^+^ expression on development of murine lupus. All cells were FACS sorted and expanded prior to adoptive transfer. Development of proteinuria and survival were measured. We found that exogenous expansion of CD4^+^CD25^+^ cells produced a population containing 70–85% CD4^+^Foxp3^+^T_regs_. Expanded T_regs_ had higher CTLA-4 and Foxp3 expression, increased *in vitro* suppression capacity, and prolonged *in vivo* survival as compared to freshly isolated cells. Adoptive transfer of expanded CD4^+^CD25^+^ T_regs_ inhibited the onset of glomerulonephritis and prolonged survival in mice. Importantly the population of T_eff_ contained within the adoptively transferred cells had reduced survival and proliferation capacity as compared to either co-transferred T_regs_ or transferred T_effs_ expanded in the absence of T_regs_. These studies demonstrate that adoptive transfer of expanded CD4^+^CD25^+^Foxp3^+^T_regs_ has the capacity to inhibit the onset of murine lupus and that this capacity is significant despite transfer of co-cultured T_eff_ cells. These data indicate that when co-expanded with regulatory T cells, exogenously activated T_effs_ from autoimmune patients may not pose a significant risk of promoting disease.

## Introduction

Systemic lupus erythematosus (SLE) is a prototypic autoimmune disease characterized by loss of tolerance to self-antigens, expansion of autoreactive lymphocytes, and immune mediated injury to multiple organ systems. The immunologic defects that permit development of SLE are incompletely understood, but as with other autoimmune diseases there is a failure to inhibit activation and expansion of autoreactive lymphocytes that escape central tolerance mechanisms. Natural regulatory T cells (T_regs_) are a thymically derived subset of CD4^+^ lymphocytes present in both humans and mice that serve an essential role in modulating the function of the immune system and in maintaining peripheral tolerance[1]–[4]. *In vitro*, CD4^+^ T_regs_ have a strong capacity to inhibit activation and expansion of both alloreactive and autoreactive T cells[3], [5]. In vivo, the T_reg_ T cell receptor (TCR) repertoire is biased towards recognition of self-antigens[6], [7] and upon activation, T_regs_ exert a potent capacity to inhibit autoreactive lymphocytes[6]–[11]. Recognition of the important role of T_regs_ in maintenance of peripheral tolerance has led to studies demonstrating an association between abnormal T_reg_ function or prevalence and the development of autoimmune disease[8], [10], [12], [13]. In several mouse systems, studies have demonstrated that therapies that restore or supplement T_reg_ function and numbers can inhibit the onset and progression of some autoimmune diseases[8], [12]–[16].

A number of studies have examined the relation between T_regs_ and SLE. In some but not all mouse models of lupus, defects in T_reg_ prevalence or function have been reported [13], [17], [18]. Similarly, a growing number of reports have described defects in the number and function of T_regs_ isolated from peripheral blood of patients with active SLE[19]–[23]. These observations suggest that T_reg_ dysfunction may be a contributing factor in the immune pathogenesis of SLE. We have previously demonstrated that supplementation of endogenous T_regs_ utilizing a highly purified, exogenously activated and expanded T_reg_ subset characterized by CD4^+^CD25^+^CD62L^HI^ expression can inhibit the onset and rate of progression of lupus in (NZBxNZW)F_1_ (B/W) mice[24]. This study suggested the possibility that augmentation of the regulatory T cell population and/or function in vivo might be beneficial. However, although these cells were able to inhibit disease, the population transferred reflected only a subset of natural T_regs_ in mice with active lupus. Some evidence suggests that different T_reg_ subsets may have unique roles in the maintenance of peripheral tolerance and thus transfer of a more comprehensive T_reg_ subset may have additional or unique therapeutic benefits[10], [25]–[27]. In addition, the intrinsic resistance of T_regs_ to exogenous expansion and the probability that a large number of T_regs_ will be required to enhance peripheral tolerance in humans makes it desirable to isolate a larger T_regs_ subset for exogenous expansion. Unfortunately, due to the lack of T_reg_ specific surface antigens, purification of a more comprehensive T_reg_ subset increases the contamination by the non-regulatory effector T cell (T_eff_) population. Despite a growing number of published purification protocols for isolating subsets of regulatory T cells, no approach to date has demonstrated the capacity to isolate the entire T_reg_ population with 100 percent specificity. Recent studies demonstrate that even the intracellular transcription factor Foxp3, considered the gold standard for identification of regulatory cells, is expressed transiently in some activated non-regulatory human T cells[28] further highlighting the difficulty in both identifying and purifying a comprehensive regulatory T cell population. Importantly, in autoimmune prone mice or humans with SLE, contamination of therapeutic T_regs_ by non-regulatory effector cells can include pathologic autoreactive lymphocytes that may have potential to exacerbate autoimmune disease if expanded and transferred back into the donor.

In this study we isolate and expand CD4^+^CD25^+^ T cells from 29-week-old lupus prone B/W mice to assess the function of this more comprehensive, but less pure T_reg_ population. We demonstrate that exogenously expanded CD4^+^CD25^+^ T_regs_ have a robust *in vitro* suppression capacity despite the inevitable presence of co-expanded non-regulatory T_effs_, many of which are contained in the CD4^+^CD25^+^CD62L^LO^ pool of recently activated T cells. Utilizing adoptive transfer studies, we demonstrate that CD4^+^CD25^+^Foxp3^+^ T_regs_ continue to divide *in vivo* and are long lived following adoptive transfer. Interestingly, while purified and *in vitro* expanded CD4^+^CD25^−^Foxp3^−^ T_eff_ cells also demonstrate the capacity to expand and survive following adoptive transfer, contaminating CD4^+^CD25^+^Foxp3^−^ T_eff_ cells expanded in co-culture with CD4^+^CD25^+^Foxp3^+^ T_regs_ cells exhibit poor survival and proliferation following adoptive transfer. Finally, we show that exogenously expanded, adoptively transferred CD4^+^CD25^+^ T cells containing a mixed population of Foxp3^+^T_regs_and co-expanded Foxp3^−^T_effs_, to lupus prone B/W mice can enhance suppression of autoreactive lymphocytes and delay the development of autoimmune disease.

## Materials and Methods

### Mice

Six-week-old B/W mice were purchased from Jackson Laboratories (Bar Harbor, Maine) and housed in the AAALAC accredited San Francisco VAMC Animal Care Facility under the supervision of a licensed veterinarian. The VAMC Institutional Animal Care Use Committee reviewed and approved all protocols utilized in this study.

### Antibodies and reagents

Monoclonal antibody (mAb) against CD4 (GK1.5), anti-Fc (2.4G2) and anti-CD3 (2C11), were purified in our lab. The GK1.5 mAb was FITC-conjugated. CD4 mAb (PerCp-Cy5.5 conjugated, RM4-5), CD62L (allophycocyanin(APC)-conjugated, MEL-14), and neutralizing antibodies to IL-10 (JES5-16E3) were purchased from BD Pharmingen (San Diego, CA). Pacific Blue-CD4 (RM4-5), Foxp3 mAb (FITC-conjugated and APC-conjugated, FJK-16s), isotype control (rat IgG2a), fixation and permeabilization buffers (catalog no. 00-5523) were purchased from eBioscience (San Diego, CA). Biotinylated goat anti-mouse IgG (catalog no. M30215), goat anti-mouse IgM (catalog no. M31515), and FITC-conjugated streptavidin (SA1001) were purchased from Caltag Laboratories (Burlingame, CA). Biotinylated rat anti-mouse C3 (11H9) was purchased from HyCult Biotechnology (The Netherlands). The anti-CD25 mAb (R-PE-conjugated, 7D4) and anti-CTLA4 mAb (R-PE-conjugated, 1B8) were purchased from Southern Biotechnology Associates, Inc (Birmingham, AL). Neutralizing antibodies to TGF-β (1D11) were purchased from R&D Systems (Minneapolis, MN). Carboxyfluoroscein succinimidyl ester (CFSE) and SNARF-1 carboxylic acid, acetate, succinimidyl ester (S22801, special packaging) was purchased from Molecular Probes/Invitrogen Life Technologies (Carlsbad, CA).

### Lymphocyte isolation

Following animal sacrifice, the spleen and cervical, axillary, inguinal, and renal lymph nodes were harvested. Single cell suspensions were prepared in DMEM with 2% fetal calf serum (FCS) by passing tissue through nylon mesh. In experiments of CFSE or SNARF-1 labeled *in vivo* cell survival, salivary glands, kidneys, lung and liver were also mechanically dissociated and lymphocytes from these tissues were isolated by ficoll gradient.

### FACS analysis and cell sorting

Cell suspensions were blocked with anti-Fc mAb prior to monoclonal antibody labeling. Lymphocytes were labeled with PerCp-Cy5.5-CD4 or FITC-CD4, PE-CD25, APC-CD62L. Foxp3 expression was measured utilizing an aliquot of cells stained with PerCp-Cy5.5-CD4 that was fixed and permeabilized, blocked with anti-Fc mAb, and stained with FITC-Foxp3 or FITC-IgG2a isotype control per manufacturers instructions (eBioscience, San Diego, CA). Combined expression of membrane bound and intracellular CTLA-4 expression was measured by first labeling surface antigen and then utilizing the fixation and permeabilization and blocking protocol defined for Foxp3 above to label intracellular antigen. All FACS analysis and sorting was performed on a FACSAria (Becton Dickinson, Mountain View, CA) instrument utilizing FACSDiva software and the purity prioritization algorithm. Purity checks of sorted cells routinely demonstrated greater than 98% purity.

### Cell culture

Purified CD4^+^CD25^+^, CD4^+^CD25^+^CD62L^HI^, CD4^+^CD25^+^CD62L^LO^, and CD4^+^CD25^−^ cells were isolated from 28–29 week old mice by FACS sorting. The frequency of T_regs_ in each sorted populations was measured by staining for Foxp3 expression in an aliquot of sorted cells. Cells were cultured utilizing the protocol previously described[29]. Briefly, purified cells were maintained at a concentration of 0.7−1×10^6^ cells/ml over an 8-day culture period in DMEM (Invitrogen) supplemented with 10% heat-inactivated fetal bovine serum (Biosource International, Camarillo, CA). 2,000 IU/ml rhIL-2 (Hoffmann-LaRoche, Nutley, NJ generously provided by the National Cancer Institute), 5 mM Hepes (Sigma-Aldrich, St. Louis, MO), NEAA, 0.5 mM sodium pyruvate, 1 mM glutaMAX (all from Invitrogen) and 50 µM β-mercaptoethanol (Sigma-Aldrich). Cells were stimulated with bead coupled anti-CD3 and anti-CD28 antibodies (Xcyte beads, Xcyte Therapeutics Inc., Seattle, WA). Following expansion, cells were separated from the Xcyte beads by centrifugation in Lympholyte-M (Cedarlane Laboratories, Burlington, NC) at 230G for 25 minutes. Aliquots of expanded cells were routinely assessed for purity by measurement of CD4^+^ and Foxp3^+^ expression and for suppressive function in a mixed lymphocyte suppression assay.

### Suppression assay

The CD4^+^CD25^−^CD62L^HI^ T cells were purified by FACS and served as responder T cells (T_resp_) in mixed lymphocyte suppression assays. CD4^+^ depleted splenocytes were irradiated at 2,000 rads and served as antigen presenting cells (APCs). Purified T_resp_ and APCs (75,000 each) were combined in 96 well U-bottom plates with soluble anti-CD3 at 4 µg/ml. T_regs_ were added to obtain a range of T_resp_∶T_reg_ ratios. Cells were cultured for 60 hours prior to addition of 1 µCi/well of [^3^H] thymidine and the assay was then harvested 12 hours later. All assays were performed in triplicate. Tritium incorporation was measured on a MicroBeta Scintillation Counter (Wallac Oy, Turku Finland). The tritium incorporation at each T_resp_∶T_reg_ was divided by incorporation in the T_resp_ only wells to calculate a suppression index (SI) for each experiment.

### In vivo survival of adoptively transferred T_regs_ and T_effs_

The *in vivo* survival of both freshly isolated and exogenously expanded T_regs_ and expanded T_effs_ was assessed in a series of experiments utilizing adoptively transferred CFSE or SNARF-1 labeled cells. All adoptive transfer studies reported in this paper utilized unmanipulated recipient mice (no irradiation or immune depletion prior to adoptive transfer). Lymphocytes from donor mice were FACS sorted and the frequency of T_regs_ and T_effs_ in freshly sorted populations was measured by FACS analysis of Foxp3 expression in an aliquot of sorted cells. Expanded cells obtained from the 8-day expansion protocol described above were also analyzed for Foxp3 expression to determine the ratio of T_regs_ to non-regulatory T_eff_ post-expansion. Fresh or expanded cells were CFSE labeled, extensively washed in sterile saline, and concentrated in 200 µl of sterile PBS in preparation for tail-vein injection to each mouse. The survival of both freshly isolated and exogenously expanded cells was measured at set time intervals from 2 to 30 days following adoptive transfer. Mice were sacrificed and single cell suspensions of lymph node and spleen were prepared. In order to detect CFSE^+^ cells that trafficked outside of the lymphatic system, lymphocytes were also recovered from salivary glands, kidneys, lung and liver. Recovered cells were stained with Pacific Blue-CD4 and APC-Foxp3 mAbs prior to FACS analysis in order to differentiate the survival and proliferation of CFSE^+^CD4^+^Foxp3^+^T_regs_ from CFSE^+^CD4^+^Foxp3^−^T_effs_. In these experiments, two types of CD4^+^Foxp3^−^T_effs_ were designated based on the culture conditions from which they were derived. The first T_eff_ population was derived from the CD4^+^CD25^+^Foxp3^−^T_effs_ (5–10% of cells) co-isolated during sorting for CD4^+^CD25^+^Foxp3^+^T_regs_. During cell culture, these contaminating T_effs_ are co-expanded in a T_reg_ predominant environment and were therefore designated as “conditioned T_effs_” (T_eff-conditioned_) to reflect potential conditioning of the T_eff_ function by T_reg_ co-culture. A second population of T_eff_ cells was generated by first depleting the CD4^+^CD25^+^ T cells (the majority of T_regs_) prior to the *in vitro* expansion of for 8-days. This expanded T_eff_ population contained <2% CD4^+^Foxp3^+^ T_reg_ cells and was therefore considered as “unconditioned” T_effs_. In specified experiments designed to assess differential survival of “unconditioned” T_eff_ cells from T_eff-conditioned_ cells following co-injection with T_regs_, mice received two tail-vein injections on the same day, one containing expanded, SNARF-1 labeled unconditioned T_effs_ and the second injection containing the expanded CFSE labeled T_regs_/T_eff-conditioned_ cells. Mice were sacrificed 4 or 5 days after injection and cell survival was assessed by FACS analysis of lymphocytes recovered from lymphatic and solid organ tissue of recipient mice (as defined above). SNARF-1^+^ and CFSE^+^ cells were then FACS sorted followed by labeling with FITC-Foxp3 or APC-Foxp3 mAbs respectively to permit differentiation of T_regs_ (CFSE^+^Foxp3^+^), T_eff-conditioned_ (CFSE^+^Foxp3^−^) cells, and “unconditioned” T_eff_ (SNARF-1^+^ Foxp3^−^) fractions. The presence of any SNARF-1^+^ Foxp3^+^ cells was also assessed.

### Adoptive cell transfer to treatment study mice

All T_reg_ and T_eff_ cells were purified from donor B/W mice age-matched to recipient mice. Expanded CD4^+^CD25^+^ T_regs_ (containing 80–85% Foxp3^+^T_regs_ and 15–20%Foxp3^−^T_eff-conditioned_) and CD4^+^CD25^−^T_eff_ controls (with <2% T_regs_) were depleted of Xcyte beads as above and extensively washed in sterile saline. A total of 6×10^6^ T_regs_ concentrated in 200 µl of sterile PBS were transferred by tail-vein injection to each mouse. Littermate mice were divided into two control groups and received either an equivalent volume of sterile PBS or 6×10^6^ expanded CD4^+^CD25^−^ T cells in 200 µl sterile PBS. A total of two separate cell sorts, exogenous expansion, and adoptive transfers were preformed in order to generate sufficient cells to treat the target of at least 20 mice per arm. The final treatment cohort contained 65 animals, comprised of 44 littermate mice divided into 15 T_reg_, 15 T_eff_, and 14 PBS recipients and 21 littermate mice divided into 7 mice per arm, for a total of 22 T_reg_ treated mice, 22 T_eff_ treated mice, and 21 PBS treated mice. All animals were 29-weeks-old at the time of adoptive transfer.

### Assessment of lupus disease activity and survival

A total of 65 mice (21–22 mice per group) were followed for the development of renal disease as measured by proteinuria and for survival. Proteinuria was measured using Uristix (Bayer Corp, Elkhart, IN). Antibodies to double-stranded DNA (anti-dsDNA Ab) were measured utilizing an ELISA previously established in our lab[30]. In a separate experiment, cohorts of 29-week old mice (5 per arm) received either T_regs_ or PBS control (as per the above protocol) and these mice were then sacrificed 8-weeks following adoptive transfer to provide renal histology. Fixed kidney tissue was stained with hematoxylin and eosin for evaluation of glomerulosclerosis and tubular damage and cryopreserved sections of kidney tissue were stained with FITC conjugated antibodies to IgG, IgM, and C3 for assessment of immune complex deposition that was scored by a blinded reader using a scoring system previously established in our laboratory[30].

### Statistical analysis

Suppression assays were performed in triplicate and the mean thymidine incorporation was assessed using the Mann-Whitney U test. Antigen expression by mean fluorescent intensity, anti-dsDNA Ab titers and renal histologic damage and immune complex deposition scores were compared by Mann-Whitney U test. Development of proteinuria, defined as ≥100 mg/dl on serial testing, and survival in treatment and control cohorts were compared by χ^2^ analysis using the Yates correction.

## Results

### Purification and expansion of CD4^+^CD25^+^T cells results in a mixed population of T_regs_ and T_effs_

**Figure 1:**
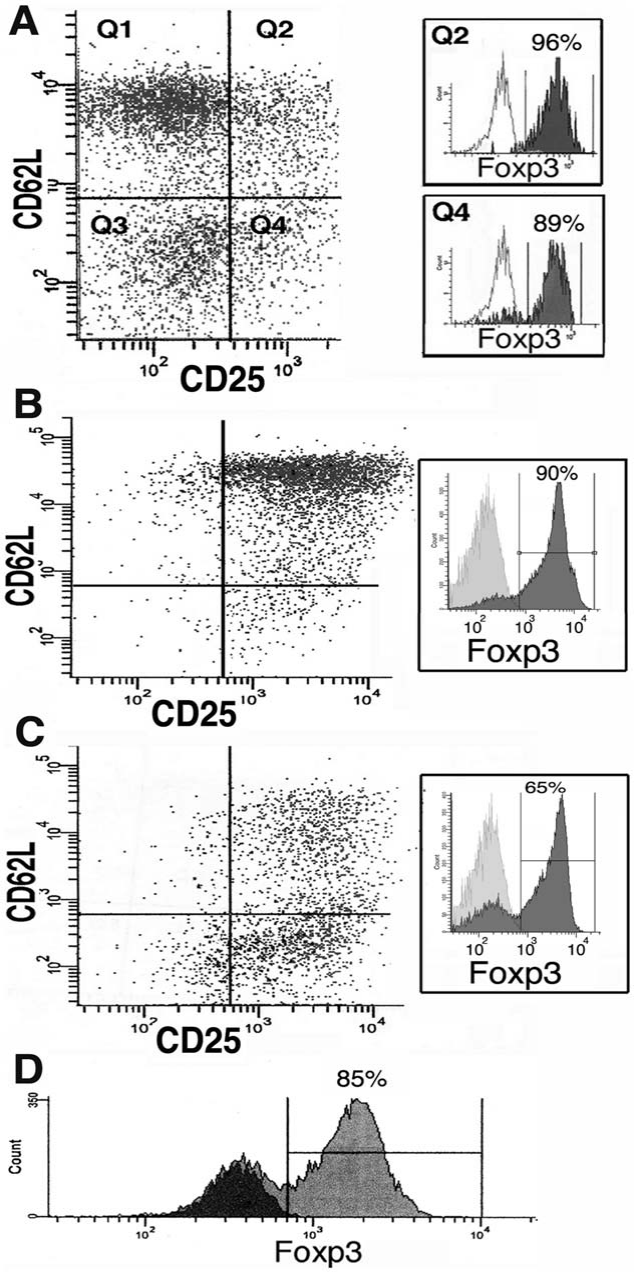
Foxp3^+^ expression in freshly isolated and expanded CD4^+^ T_reg_ cells. A) Foxp3 expression in fresh CD25^+^CD62L^HI^ cells (Q2, 96%) versus CD25^+^CD62L^LO^ cells (Q4, 89%). Isotype control antibody shown for each histogram. B) Expanded Q2 cells demonstrating maintenance of CD62L^HI^ and Foxp3^+^expression (90%). C) Expanded Q4 cells demonstrating up-regulation of CD62L^HI^ on 33% of cells and a mixture of 65% Foxp3^+^T_regs_ and 35% Foxp3^−^ T_eff_ cells post-expansion. D) Foxp3^+^ expression (grey histogram, 85% of cells) following expansion of the entire CD4^+^CD25^+^ T cell subset. Isotype control shown in black histogram.

In B/W mice with active lupus, the frequency of natural T_regs_, as measured by Foxp3^+^ expression, was 96±2% in the cells identified by CD4^+^CD25^+^CD62L^HI^ expression (Figure 1A). The CD4^+^CD25^+^CD62L^LO^ population contained both Foxp3^+^T_regs_ and a significant population of recently activated Foxp3^−^T_eff_, such that a typical sort of this quadrant produced a population containing 85–92% Foxp3^+^T_regs_ and 8–15% Foxp3^−^ T_effs_ (Figure 1A, 36-week old B/W mouse with early proteinuria). *In vitro* expansion of cells over an 8-day period increased the relative frequency of Foxp3^−^ cells due to the more rapid proliferation of T_effs_ as compared to T_regs_ during culture. The CD4^+^CD25^+^CD62L^HI^ cells maintain this surface phenotype during expansion and produce an expanded population that is ∼85–90% Foxp3^+^ post-expansion (Figure 1B). Expansion of the CD62L^LO^ subset produced cells with mixed surface phenotype (33% express CD62L^HI^) and a reduced Foxp3^+^ purity (65–85%) due to the higher initial T_eff_ prevalence (Figure 1C). On average, culture of the entire CD4^+^CD25^+^ cell population produced a population that expanded 30 to 40-fold and was 70–85% CD4^+^Foxp3^+^ (Figure 1D).

### In vitro T_reg_ suppressor function is similar between subsets of CD25^+^ T_regs_ and this function is enhanced by in vitro activation and expansion

**Figure 2:**
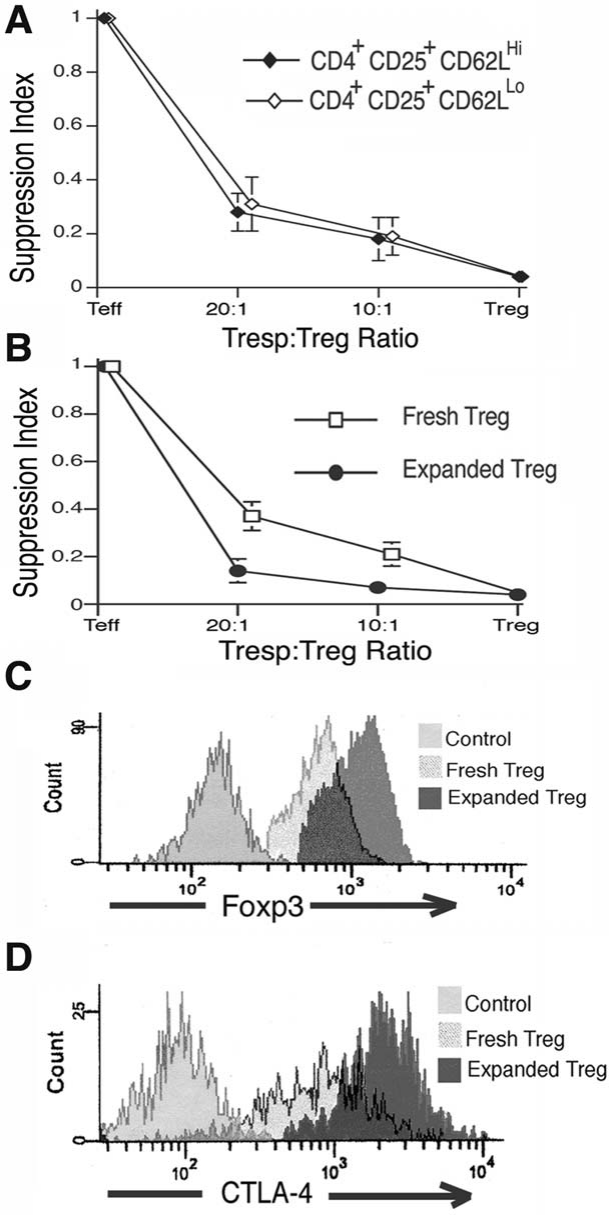
CD4^+^CD25^+^T_reg_ function and phenotype is enhanced by exogenous expansion. A) Freshly isolated CD62L^HI^ and CD62L^LO^ fractions of CD4^+^CD25^+^T_regs_ demonstrate equivalent suppression capacity *in vitro* as measured by mixed lymphocyte suppression assay (p≥0.25 for each cell ratio, 3–6 mice per group). The Suppression Index measures the capacity of T_regs_ to suppress T_resp_ proliferation (as measured by decreased tritium-labeled thymidine incorporation) at different T_resp_∶T_reg_ ratios divided by the maximal T_resp_ proliferation in the absence of T_regs_ B) *Ex vivo* expansion of CD4^+^CD25^+^ T_regs_ significantly enhances the ability of these cells to suppress proliferation of T_resp_ cells derived from 29-week-old B/W mice (p≤0.04 for each cell ratio, 6 mice per group). C–D) Foxp3^+^ and CTLA-4 (surface and intracellular) expression in exogenously expanded T_regs_ were significantly greater than in freshly isolated T_regs_ as measured by mean-fluorescent intensity (p = 0.007, p<0.001 respectively, 3–6 mice per group).

We previously demonstrated that the *in vitro* suppressive capacity of the CD4^+^CD25^+^CD62L^HI^ T_reg_ fraction is not affected by the age or disease state of donor B/W mice and is equivalent to that of similar cells from non-autoimmune prone BALB/c mice[24]. In this study, the CD4^+^CD25^+^CD62L^LO^ T_regs_ from B/W mice with active disease were isolated by FACS sorting and the suppressor capacity was compared to the CD4^+^CD25^+^CD62L^HI^ T_reg_ fraction. Freshly isolated cells demonstrated equivalent suppression between these CD62L^HI^ and CD62L^LO^ T_reg_ subsets (Figure 2A, p≥0.25 for each cell ratio, 3–6 mice per group).

To evaluate the effect of *ex vivo* expansion on suppressive function, we isolated the entire CD4^+^CD25^+^ T cell population and measured *in vitro* suppressive function before and after expansion. Despite the presence of 15–20% co-expanded T_effs_, the expanded CD4^+^CD25^+^T_regs_ demonstrated significantly enhanced suppression capacity across the range of T_resp_∶T_reg_ ratios tested as compared to freshly isolated CD4^+^CD25^+^ cells (Figure 2B, p≤0.04 for each cell ratio, 6 mice per group). This enhanced T_reg_ suppressor function following exogenous expansion is consistent with prior reports of T_reg_ function in other murine models and with our previous findings with the CD4^+^CD25^+^CD62L^HI^T_reg_ fraction in B/W mice[24], [31]. Because levels of Foxp3^+^ and CTLA-4 expression in T_regs_ have been correlated with suppressor function of these cells and these antigens are also known to be up-regulated by T_reg_ activation, we measured their expression in freshly isolated and exogenously expanded T_regs_ from young and old B/W mice by FACS. Levels of Foxp3 or CTLA-4 expression in freshly isolated T_regs_ as measured by mean fluorescent intensity (MFI) did not vary with mouse age or organ distribution (data not shown). However, consistent with prior reports in other mouse strains[29], [31] there was a marked increase in both Foxp3^+^ and CTLA-4 expression in B/W T_regs_ following exogenous expansion (Figure 2C–D, p<0.01 for both, 3–6 mice per group).

### Exogenous expansion significantly enhances in vivo survival of T_regs_, but not of co-cultured T_eff_

**Figure 3:**
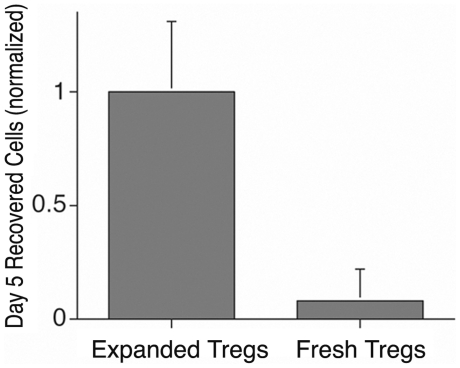
Enhanced *in vivo* survival of expanded T_regs_ as compared to freshly isolated T_regs_ following adoptively transfer. Bar graph demonstrates that the number of exogenously expanded CFSE^+^CD4^+^Foxp3^+^T_regs_ recovered from lymphatic and solid organs 5 days after adoptive transfer (adjusted for the variability in total cell recovery from each recipient mouse) is significantly greater than that for freshly isolated and adoptively transferred T_regs_ (p<0.01, 3 mice per group).

We assessed the *in vivo* survival of freshly isolated and exogenously expanded T_regs_ and expanded T_eff_ cells in a series of transfer experiments utilizing mixed populations of expanded T_regs_/T_eff-conditioned_, and T_effs_. Prior studies have shown that T_reg_ interactions with T_effs_ can induce long term T_eff_ anergy[32], [33], but whether this *in vitro* interaction influences the *in vivo* survival and proliferation of contaminating T_effs_ within the expanded T_reg_ population is unknown. The *in vivo* survival of freshly isolated, adoptively transferred CD4^+^CD25^+^T_regs_ was first compared to that of exogenously expanded T_regs_. Cohorts of 3 mice each received aliquots of sorted fresh or expanded CFSE labeled cells, each containing 1.6×10^6^ Foxp3^+^ T_regs_. Five days following transfer, recipient mice were sacrificed and the lymphocyte recovery from lymph nodes, spleen and tissues (liver, kidney, lung, salivary glands) was measured. An aliquot of cells was then stained for Foxp3 prior to FACS analysis. The number of CFSE^+^Foxp3^+^ cells (as a percentage of all cells in the aliquot) and the total cell recovery from each mouse was then used to estimate the T_reg_ survival in each mouse. As shown in Figure 3, *in vivo* survival of exogenously expanded, adoptively transferred T_regs_ at day 5 is significantly enhanced as compared to that of freshly isolated cells (p<0.01). Freshly isolated, adoptively transferred CFSE^+^T_regs_ could no longer be consistently detected by FACS when mice were examined 10 days post transfer.

**Figure 4:**
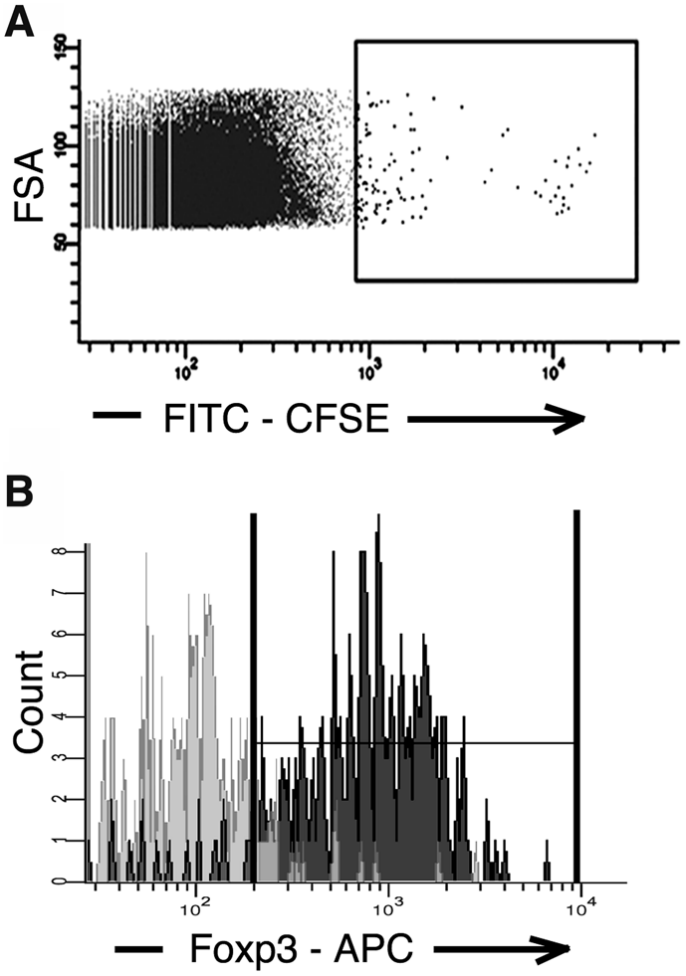
*In vivo* survival and proliferation of expanded T_regs_ detected 30 days following adoptive transfer. A) A small population of CFSE^+^ labeled cells could be detected and FACS sorted at 30 days from a pool of lymphocyte isolation from lymphatic, splenic, salivary gland, lung, liver and kidney tissue (FITC bright sort gate shown containing ∼0.2% of CD4^+^ T cells). B) Characteristic Foxp3 staining of sorted FITC bright cells demonstrating >90% of these cells are Foxp3^+^ (dark grey histogram), consistent with these cells being derived from the adoptively transferred CFSE labeled, Foxp3^+^T_regs_ and not FACS artifact (isotype control shown in light grey histogram, 3 mice cohort).

The long-term *in vivo* survival of exogenously expanded T_regs_ was then assessed by following 10×10^6^ cells for up to 30 days following transfer (3 mouse cohort). As shown in Figure 4, a small population of CFSE^+^ positive cells could be detected at 30 days (Figure 4A, ∼0.2% of CD4^+^ T cells). To ensure these rare cells were actually the adoptively transferred CFSE labeled T_regs_ and not just very large cells or cellular doublets with bright autofluorescence, the CFSE bright population was FACS sorted and stained for Foxp3 expression. As shown in Figure 4B, Foxp3 was detected in >90% of CFSE^+^ cells consistent with long term survival and proliferation of exogenously expanded, adoptively transferred T_reg_.

**Figure 5:**
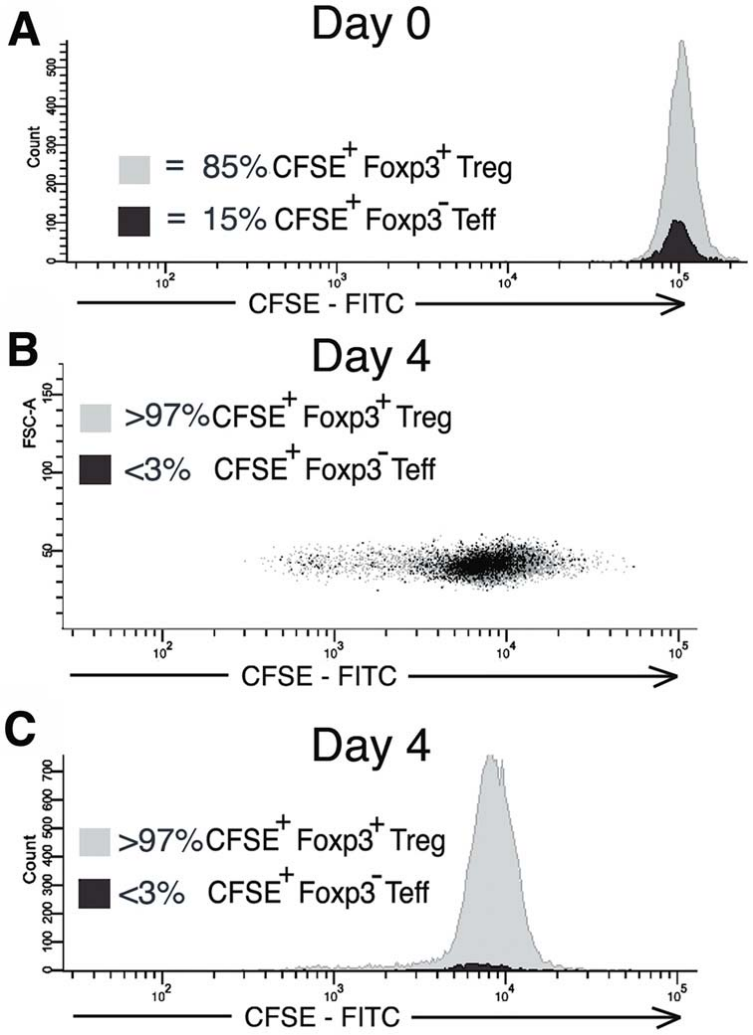
CD4^+^Foxp3^−^T_eff-conditioned_ demonstrate impaired *in vivo* survival and proliferation following adoptive transfer. At each time point, an aliquot of CFSE labeled cells was fixed and stained with Pacific-Blue Foxp3 to permit measurement of the ratio T_regs_ and T_eff-conditioned_. A) Baseline FITC-CFSE fluorescence of expanded CD4^+^CD25^+^ lymphocytes just prior to adoptive transfer. Foxp3 stain demonstrates this exogenous expanded population containing 85% Foxp3^+^T_regs_ and 15% Foxp3^−^T_eff-conditioined_ (contaminating T_eff_ cells contained within the expanded T_regs_ population) at the time of transfer. B–C) Dot plot and histogram of the FITC-CFSE fluorescent intensity of both Foxp3^+^ T_regs_ and Foxp3^−^ T_eff-conditioned_ cells recovered from lymphatic and solid organ tissue 4 days after adoptive transfer. Panels B–C demonstrate a significantly reduced survival and proliferation of T_eff-conditioned_ as compared to T_regs_ such that T_eff-conditioned_ comprise <3% of the recovered CFSE^+^ population. Data reflects typical finding from 6 separate experiments.

**Figure 6:**
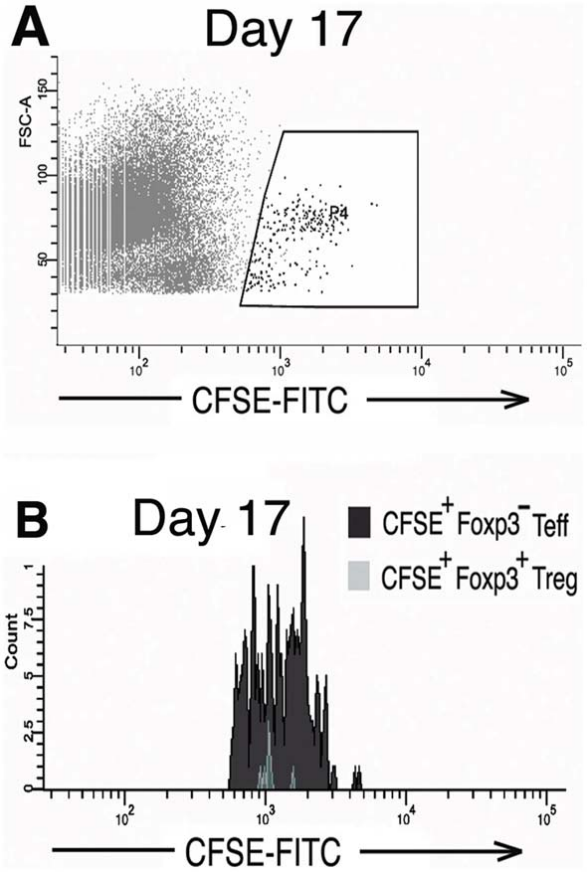
Persistent *in vivo* survival and proliferation of CD4^+^Foxp3^−^ T_effs_ when these cells are first expanded *in vitro* under T_reg_ depleted culture conditions (expanded, “unconditioned” T_effs_). A) Survival and division of CFSE labeled, unconditioned CD4^+^Foxp3^−^ T_effs_ could be detected up to 17 days following adoptive transfer at which time the FITC intensity of CFSE labeled cells approached the auto-fluorescent baseline. Dot plot at day 17 demonstrating the sorting gate for FITC-bright, CFSE^+^ cells. B) Cells sorted from the gate in Panel A are >98% Foxp3^−^ consistent with the adoptively transferred T_effs_ (characteristic findings from 3 mice).

**Figure 7:**
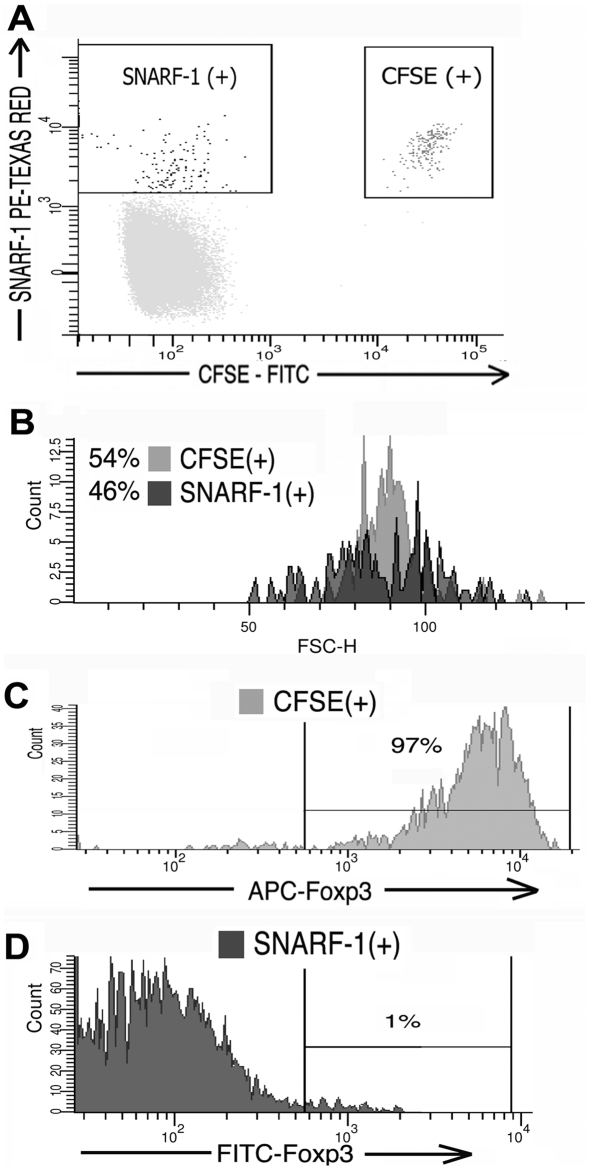
*In vivo* survival and proliferation of expanded T_regs_ and T_effs_ (independently injected into the same mouse) indicating impaired survival of T_eff-conditioned_ is not intrinsic to all transferred T_effs_, but instead due to co-culture with T_regs_. A) CFSE^+^ and SNARF-1 cells recovered four days following independent injections of expanded CFSE^+^ CD4^+^CD25^+^ cells (containing T_regs_ and T_eff-conditioned_) and expanded SNARF-1^+^ CD4^+^CD25^−^ T_effs_ (unconditioned T_effs_) demonstrating approximately equal survival of both populations (∼0.4% of recovered lymphocytes each). The ratio of injected cells on Day 0 was 45% T_regs_, 10% T_eff-conditioned_, 45% unconditioned T_effs_. B) Histogram of recovered CFSE^+^ and SNARF-1^+^ cells at day 4 demonstrate persistence of “unconditioned” T_effs_ (SNARF-1^+^ cells) equivalent to survival of the CFSE^+^T_reg_/T_eff-conditioned_ mix, a finding not observed when only T_regs_ and T_eff-conditioned_ are co-transferred (as shown in Figure 5). C–D) Foxp3 analysis of sorted cells demonstrating that surviving CFSE^+^ cells are T_regs_ (97% Foxp3^+^) and SNARF-1^+^ cells are “unconditioned” T_effs_ (Foxp3^−^), indicating impaired survival and proliferation of transferred T_eff-conditioned_ is a consequence of co-culture with T_reg_ during exogenous expansion (characteristic findings from 5 mice).

To assess the survival of contaminating, co-transferred T_eff_ cells contained within the expanded T_reg_ population, cohorts of 3 mice received expanded CD4^+^CD25^+^ cells derived from the high purity T_reg_ culture that contained 85% CD4^+^Foxp3^+^T_regs_ and 15% contaminating CD4^+^Foxp3^−^T_eff-conditioned_ (Figure 5A). Two to eight days following transfer mice were sacrificed and the lymph node, spleen, and tissue infiltrating lymphocytes were FACS analyzed for CFSE and Foxp3. As demonstrated in Figure 5B–C, the frequency of CFSE^+^Foxp3^−^T_eff-conditioned_ relative to CFSE^+^Foxp3^+^T_regs_ rapidly declined such that by day 4 the T_eff-conditioned_ comprised <3% of the transferred cells (an 80% decline) with no evidence of significant T_eff-conditioned_ division by CFSE dilution. To determine if this decline in T_effs_ prevalence was a result of conditioning during co-culture with T_regs_ or an intrinsic property of all exogenously expanded, adoptively transferred T_effs_, *in vivo* survival of T_effs_ expanded in the absence of T_regs_ was assessed (“unconditioned” T_effs_ generated from expansion of CD25-depleted CD4^+^ T cells. Expanded cells were 98% CD4^+^Foxp3^−^T_effs_). In the first experiment, survival and division of 5×10^6^ transferred CFSE labeled, Foxp3^−^ unconditioned T_effs_ could be detected by FACS up to seventeen days post-transfer, at which time CFSE fluorescent intensity approached the autofluorescence baseline (Figure 6). The relative survival of unconditioned T_effs_ was then compared to survival of expanded T_regs_ following transfer of these two populations into the same mouse. Mice received injections of 3.45×10^6^ expanded, CFSE labeled CD4^+^CD25^+^ cells (83% T_regs_ and 17% T_eff-conditioned_ by Foxp3 stain) and a second injection of 2.86×10^6^ expanded, SNARF-1 labeled CD4^+^CD25^−^ T_effs_ (“unconditioned” T_effs_). Initial ratio of injected cells was 45% T_regs_, 10% T_eff-conditioned_, and 45% unconditioned T_effs._ Four to five days following transfer, analysis of surviving CFSE^+^ and SNARF-1 cells demonstrated a persistence of T_regs_ and T_effs_, but not T_eff-conditioned_. Figure 7A–B show a characteristic result at day 4 at which time approximately equal numbers of CSFE and SNARF-1 labeled cells could be recovered from the lymphatic system and organs (0.4% of total lymphocytes each, ratio of sorted CFSE to SNARF-1 is 54% to 46%). Foxp3 analysis of recovered CFSE^+^ cells demonstrates that these cells are comprised almost entirely of T_regs_ (Figure 7C, 97% of surviving CFSE^+^ cells are Foxp3^+^T_regs_, 3% Foxp3^−^T_eff-conditioned_). Recovered SNARF-1^+^ were unconditioned T_effs_ as expected (Figure 7D, 99% Foxp3^−^) (characteristic findings from 5 mice). This data is consistent with evidence in Figures 5 and 6 demonstrating that impaired T_eff-conditioned_ survival is a consequence of co-culture with T_regs_ and not an intrinsic characteristic of all adoptively transferred T_effs_.

### Adoptive transfer of exogenously expanded CD4^+^CD25^+^ T_regs_ to pre-nephritis mice slows the progression to proteinuria and prolongs survival

**Figure 8:**
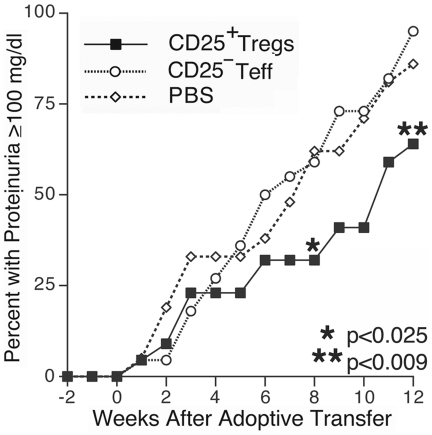
Inhibition of proteinuria in T_reg_ treated mice. Mice receiving adoptively transferred T_regs_ had significantly reduced progression to proteinuria ≥100 mg/dl over the 12 weeks following transfer as compared to either PBS or CD4^+^CD25^−^ T cell control groups (p≤0.025 starting week 8, n = 22 for T_reg_ and T_eff_ groups, n = 21 for PBS group).

**Figure 9:**
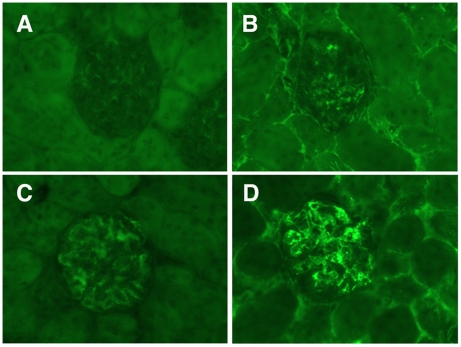
Inhibition of immune complex renal damage in T_reg_ treated mice. Typical kidney sections demonstrating less severe IgG and IgM deposition in mice receiving adoptive T_reg_ transfer (Figure 6A–B respectively) as compared to mice receiving either control therapy (T_eff_ control mouse, Figure 6C–D).

**Figure 10:**
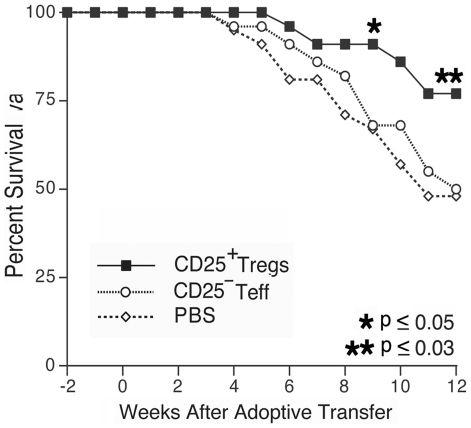
Prolonged survival in T_reg_ treated mice. Mice receiving adoptive transfer of T_regs_ had significantly prolonged survival first evident 9 weeks after transfer (p<0.05 at week 9) as compared to either control group.

To determine if adoptive transfer of a mixed population of exogenously expanded CD4^+^CD25^+^ T_regs_ can suppress murine lupus, we transferred cells to a large cohort of 29 week-old B/W mice without clinical disease. Treatment mice received 6×10^6^ T_regs_ via tail vein injection, based on our prior ability to delay disease onset utilizing this number of CD62L^HI^ T_regs_. Control mice received either an equivalent number of exogenous expanded CD4^+^CD25^−^ cells or PBS. Following adoptive transfer, mice in the active treatment group had delayed progression to significant renal disease, such that eight weeks after cell transfer, only 32% of animals in the treatment group had developed proteinuria compared to 59% in the CD4^+^CD25^−^ T_eff_ mice and 62% in the PBS control group (Figure 8, p<0.025 for both comparisons. Dataset S1 contains proteinuria and survival data). Mice sacrificed for renal pathology and immunohistochemical evaluation at 8 weeks following the initiation of therapy exhibited diminished immune complex deposition among those that received T_regs_ (Dataset S2 contains proteinuria data). Indirect immunfluorescence demonstrated that kidneys from T_reg_ treated mice (Figure 9, A–B) had less IgG and IgM immune complex deposition as compared to control treated mice (Figure 9, C–D, p≤0.05 for both comparisons. Differences in C3 deposition and renal damage pathologic scoring did not reach statistical significance). The prevalence of proteinuria in T_reg_ treated mice remained significantly lower than either control group up to 12 weeks following the adoptive transfer (p≤0.008 for both comparisons). Inhibition of renal disease in T_reg_ recipients correlated with an improved survival first evident 9 weeks following adoptive transfer (Figure 10), p<0.05 T_reg_ vs. either control. This survival advantage in the T_reg_ recipients remained significant during the 12 weeks following transfer (p≤0.026). In mice followed beyond the 12-week experiment (15 Tr_eg_, 15 T_eff_, 14 PBS), it was observed that the majority of mice in each treatment arm eventually developed proteinuria, but the delay in disease onset and prolonged survival of T_reg_ recipient mice remained evident to 24 weeks following therapy (Dataset S1). At baseline, all mice in each treatment arm had anti-dsDNA antibody titers below the detection threshold. Significant variability in autoantibody titer was observed among mice in each group during follow-up and the difference between groups did not reach statistical significance.

## Discussion

In this report we expand on our prior work demonstrating the ability of exogenously expanded, adoptively transferred T_regs_ to inhibit the development of murine lupus. We demonstrate that exogenous expansion and transfer of T_regs_ contained within the population of CD4^+^CD25^+^ T cells suppresses the onset of glomerulonephritis and prolongs survival in a murine model of lupus. Importantly, the co-transfer of a population of co-purified/co-expanded T_effs_ did not negate this therapeutic benefit in mice. These results underscore the remarkable ability of T_regs_ to suppress autoreactive immune responses. They also address an important concern with respect to the therapeutic potential of this approach in terms of the risk of simultaneous transfer of activated, autoreactive and pathogenic T_effs_ to autoimmune recipients. In this study we found that co-expanded and transferred T_eff_, that are present as a consequence of sorting and expanding T_regs_ without the availability of T_reg_ specific cell surface antigens, did not off-set the therapeutic benefit of exogenously expanded, adoptively transferred T_regs_. In addition, we tracked the survival and proliferation of non-regulatory/Foxp3^−^ cells co-transferred with the T_reg_ population and found T_effs_ expanded in the presence of T_regs_ do not persist *in vivo*, particularly compared to transferred Foxp3^+^ T_regs_. The poor survival and lack of division of the co-cultured Foxp3^−^T_effs_ produced under our *in vitro* expansion conditions indicates these cells are unlikely to have the capacity to contribute either to the disease suppression (as iT_regs_) or adversely to the pool of self-reactive T_effs_. This observation is consistent with the evidence that activated T_regs_ produce a significant amount of TGF-β and that both human and mouse T_effs_ activated in the presence of TGF-β develop an anergic phenotype and under some conditions can be converted to an induced suppressor cell (iT_reg_)[32], [34], [35]. This finding is an important first step in considering the possibility of utilizing T_reg_ as a therapeutic approach in humans.

The ability of adoptively transferred T_regs_ to suppress murine lupus also raises a number of questions about how exogenously expanded cells function to suppress disease that is not controlled by the endogenous T_reg_ population. We previously demonstrated that B/W mice with active lupus have a significantly expanded T_reg_ population that is equal to or exceeds the population found in non-autoimmune prone animals[24]. Yet it appears that this T_reg_ expansion is insufficient to control the proliferation and function of autoreactive lymphocytes in lupus. One possible explanation for this failure is that the T_reg_ expansion is simply insufficient to suppress the number of autoreactive lymphocytes that occur in lupus. Another possibility is that the endogenous T_regs_ do not have proper function in vivo. Our extensive *in vitro* evaluation of purified B/W T_regs_ suggest that these cells do not have a defect in their ability to suppress autologous T cell proliferation, however the true functional capacity of these cells in vivo is not known. Importantly, while the *in vitro* suppressive function of freshly isolated B/W T_regs_ is equivalent to T_regs_ isolated from non-autoimmune prone BALB/c mice, this function can nonetheless be significantly enhanced by *in vitro* activation. Consistent with observations in other murine models, expanded B/W T_regs_ also have higher Foxp3^+^ and CTLA-4 expression. In addition, the expanded T_regs_ demonstrate significantly enhanced *in vivo* survival as compared to freshly isolated cells. These data suggest a basis for the efficacy of ex-vivo expanded autologous T_regs_ in suppressing disease in lupus prone mice. In some autoimmunity models the T_effs_ are resistant to T_reg_ suppression *in vitro* and/or in vivo[17], [36]. For this study we utilized T_eff_ from 29-week-old mice that on average had developed antinuclear antibodies and low titers of dsDNA antibodies, but had not yet progressed to clinically active lupus. In this critical stage of disease evolution we did not observe resistance of T_eff_ cells to CD4^+^CD25^+^ T_reg_ mediated *in vitro* suppression. It is known that T_regs_ must undergo activation in the peripheral circulation in order to achieve a suppressor phenotype. It is thus possible that the *in vitro* suppression assay is insensitive to a heterogeneous T_reg_ activation state due to the dominant suppression capacity of those T_regs_ that have already become activated. The enhanced *in vitro* and likely *in vivo* function of T_regs_ we observe following *in vitro* activation likely reflects at least in part the suppressive function of a more homogenously activated T_reg_ population. Additional studies will be required to better characterize the factors responsible for the enhanced suppressor capacity of these expanded cells. In this study we did not attempt to compare disease suppression by supplementation of freshly isolated T_regs_ based on several observations. The protocol to isolate highly enriched T_regs_ results in a total recovery of fresh T_regs_ from each donor mouse of only 400,000 to 800,000 per mouse and it would therefore require an impractical average of 8 donor mice for each treatment mouse tested. In addition, the *in vivo* survival of freshly isolated, CFSE labeled T_regs_ demonstrated these cells had very poor survival relative to expanded cells decreasing the potential biologic impact of transferred freshly isolated cells.

Despite inhibition of lupus nephritis and prolonged survival, the majority of mice receiving T_reg_ transfer progressed to active disease during long-term follow-up. This pattern of inhibiting disease onset, but not the final disease severity with a single adoptive transfer is similar to the benefit observed when a more restricted population of CD4^+^CD25^+^CD62L^HI^ T_regs_ was adoptively transferred. While not compared directly in a single treatment study, no significant differences in either the size or duration of the treatment response is evident between mice receiving equal numbers of expanded CD4^+^CD25^+^CD62L^HI^ or CD4^+^CD25^+^ T_regs_. Future studies utilizing more sensitive biomarkers of disease activity than proteinuria, antibody formation, and survival will be required to clarify the role of different T_reg_ subsets in disease suppression. Multiple factors may contribute to the duration of therapeutic benefit of a single adoptive transfer. Limitations on the ability to track adoptively transferred T_regs_ in our B/W mice leave unresolved the question of how long transferred cells survive and continue to exert a suppressor phenotype. Due to their wide distribution to both lymphatics and solid organ tissue, it is not possible to specify the exact number of surviving cells at a given time point following transfer. In the current study we detected transferred cells surviving and proliferating to 30 days, but the absolute number of recovered cells is small and may reflect a diminishing survival with time. From our sampling of major lymph nodes, spleen, and solid organs we estimate that ∼10% of cells persist 5–7 days post transfer, ∼1–3% persist 14 days post transfer, and <1% demonstrate long term survival to our detection threshold of 30 days. Whether the delay in disease onset is due to transient non-specific bystander suppression effect from the large number of activated T_regs_ transferred or from the smaller number of long lived, potentially antigen specific T_regs_ is unclear. While further studies will be required to expand the understanding of both the mechanisms of action and therapeutic potential of adoptively transferred T_regs_ in the treatment of SLE, this study supports the potential benefits of augmenting T_reg_ number or function as a novel therapeutic approach to the treatment of this disease.

In conclusion, our results demonstrate that exogenous expansion and adoptive transfer of the regulatory T cell subset defined by CD4 and CD25 expression produces a population of highly suppressive, long-lived T_regs_ capable of delaying the onset of glomerulonephritis in a murine model of lupus. Co-culture and adoptive transfer of a small population of contaminating, potentially autoreactive T_effs_ does not exacerbate autoimmune disease or limit the potential of T_regs_ to suppress disease, in part due to the short *in vivo* survival time and anergic phenotype of these co-cultured cells.

## Supporting Information

Dataset S1 Proteinuria and Survival Data. Serial proteinuria and survival in treatment and control mice. Mice received CD4^+^CD25^+^T_regs_, CD4^+^CD25^−^T_effectors_ control cells, or PBS. Proteinuria score is recorded as 0–4 as measured by urine dipstick. Mouse deaths noted by X marks.
(0.04 MB XLS)

Dataset S2 Serial proteinuria in renal histology mice. Mice were sacrificed 8-weeks after adoptive transfer of CD4^+^CD25^+^T_regs_ or PBS. Proteinuria score is recorded as 0–4 as measured by urine dipstick.
(0.02 MB XLS)
